# Functional Scaffolds for Bone Tissue Regeneration: A Comprehensive Review of Materials, Methods, and Future Directions

**DOI:** 10.3390/jfb15100280

**Published:** 2024-09-25

**Authors:** Emily Ann Todd, Nicholas A. Mirsky, Bruno Luís Graciliano Silva, Ankita Raja Shinde, Aris R. L. Arakelians, Vasudev Vivekanand Nayak, Rosemary Adriana Chiérici Marcantonio, Nikhil Gupta, Lukasz Witek, Paulo G. Coelho

**Affiliations:** 1 University of Miami Miller School of Medicine, Miami, FL 33136, USA; 2 Biomaterials Division, NYU Dentistry, New York, NY 10010, USA; 3 Department of Diagnosis and Surgery, School of Dentistry of Araraquara, São Paulo State University (UNESP), Araraquara 01049-010, Brazil; 4 Department of Mechanical and Aerospace Engineering, NYU Tandon School of Engineering, Brooklyn, NY 11201, USA; 5 Division of Plastic Surgery, DeWitt Daughtry Family Department of Surgery, University of Miami Miller School of Medicine, Miami, FL 33136, USA; 6 Department of Biochemistry and Molecular Biology, University of Miami Miller School of Medicine, Miami, FL 33136, USA; 7 Department of Biomedical Engineering, NYU Tandon School of Engineering, Brooklyn, NY 11201, USA; 8 Hansjörg Wyss Department of Plastic Surgery, NYU Grossman School of Medicine, New York, NY 10016, USA

**Keywords:** functionalization, bone tissue regeneration, scaffolds, osseous defects, 3D printing

## Abstract

Bone tissue regeneration is a rapidly evolving field aimed at the development of biocompatible materials and devices, such as scaffolds, to treat diseased and damaged osseous tissue. Functional scaffolds maintain structural integrity and provide mechanical support at the defect site during the healing process, while simultaneously enabling or improving regeneration through amplified cellular cues between the scaffold and native tissues. Ample research on functionalization has been conducted to improve scaffold–host tissue interaction, including fabrication techniques, biomaterial selection, scaffold surface modifications, integration of bioactive molecular additives, and post-processing modifications. Each of these methods plays a crucial role in enabling scaffolds to not only support but actively participate in the healing and regeneration process in bone and joint surgery. This review provides a state-of-the-art, comprehensive overview of the functionalization of scaffold-based strategies used in tissue engineering, specifically for bone regeneration. Critical issues and obstacles are highlighted, applications and advances are described, and future directions are identified.

## 1. Introduction

**Figure 1:**
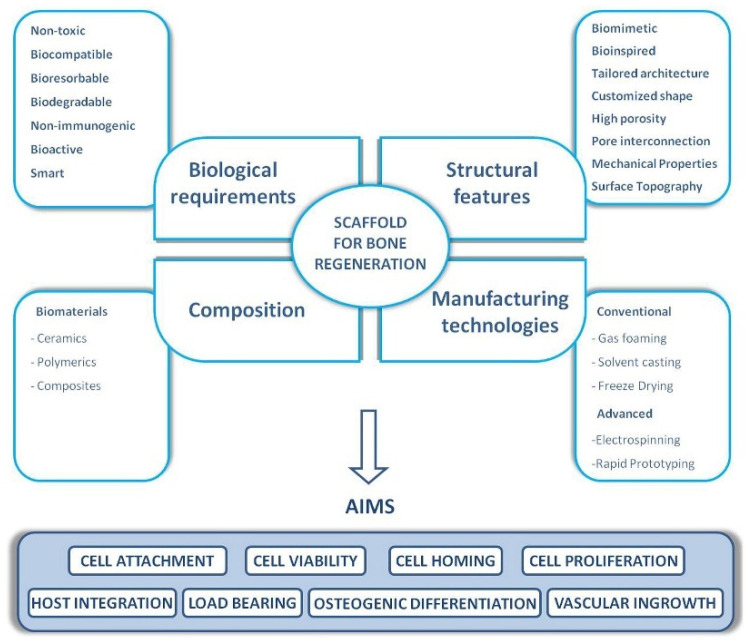
Properties that an ideal scaffold should display for bone tissue engineering applications. Reprinted with permission from ref. [11]. Copyright 2017 Elsevier Ltd.

Various factors, including but not limited to cancer, trauma, injuries, systemic disorders (i.e., genetic conditions (e.g., osteogenesis imperfecta)), and different diseases (i.e., osteoarthritis, osteoporosis), can cause tissue deficiencies [1]. Numerous advancements have been reported in the field of biomaterials and their manufacturing modalities that aim to repair or restore tissue damage [1,2]. The material selected for scaffold fabrication and post-processing should be suitable for the desired biochemical and physical properties for in vivo success, as well as demonstrate compatibility with the specific manufacturing technique [2]. An ideally developed material for tissue engineering must exhibit three major fundamental characteristics: (i) mechanical stability [1,3]; (ii) biocompatibility and/or bioactivity [1,4]; and (iii) biodegradability [1,5] (Figure 1).

Apart from the commonly utilized metallic-based tissue engineering devices, the field of biomaterials that has been extensively studied over the past few decades also includes ceramic- and polymeric-based materials [1,6]. With the latter, the organic materials are composed of long chains of atoms joined by natural or synthetic covalent bonds [1,7]. For example, polymers are widely used with or without the addition of cellular components or biological mediators in the regeneration of dental structures, periodontal supporting structures, maxillary sinuses, temporomandibular joints, and salivary glands [1]. Most scaffolds studied for bone regeneration include natural polymers such as chitosan, fibrin, hyaluronic acid (HA), and collagen (COL), as well as synthetic polymers such as polylactic acid (PLA), and polycaprolactone (PCL). Additionally, bioactive ceramics, including coralline, hydroxyapatite (HAp), tricalcium phosphate (TCP), bioactive glass, and calcium silicate, have been explored as viable solutions. Hybrid combinations, such as copolymers, polymer–polymer blends, or polymer–ceramic composites, have also been investigated [8,9], and most recently, hydrogel-based scaffolds have been gaining attention due to their ability to create well-defined 3D tissue analogs resembling the native extracellular environment. Hydrogels can be molded into various shapes and sizes under cytocompatible conditions, containing a low dry mass (1–20%) that minimizes inflammation and foreign body reactions during degradation. These attributes make hydrogels promising candidates for bone regeneration [8,10].

As previously demonstrated, a wide range of biomaterials can be used preclinically to provide osteogenesis, osteoinduction, and osteoconduction for bone regeneration; however, few have been tested in clinical studies [8,12,13]. The preclinical evaluation of the selected biomaterials is a mandatory step before starting clinical trials, according to regulatory agencies [8]. However, as bone tissue engineering progresses/transitions toward clinical applications, it becomes crucial to demonstrate the therapeutic efficacy of these innovative scaffold designs [8]. In response to the growing demand for patients, as well as the significant reduction in their age range, surgeons have been adopting new surgical techniques and designs in bone and joint reconstructive procedures [14]. In the context of surgeries focused on bone tissue reconstruction, preventing potential complications is closely associated with the design of scaffolds that precisely fit three-dimensionally into anatomical defects [15]. Given the intricate nature of the osteochondral junction, multilayer scaffolds with distinct biological and mechanical characteristics have been used in cartilage tissue engineering, with the goal of fostering simultaneous growth of cartilage and bone layers within a single, functionalized, integrated scaffold [15].

Functionalization involves modifying the scaffold to enhance its interaction with native biological tissues, promote desired cellular responses, and facilitate the regeneration process. This can be achieved through a variety of methods that tailor the scaffold’s physical, chemical, and biological properties. The primary goal is to create an environment that closely mimics the natural ECM, thereby improving the scaffold’s performance in clinical applications. Key methods employed to functionalize scaffolds include the incorporation of bioactive molecules, the seeding of stem cells to enhance tissue regeneration, and the influence of microarchitectural design and surface topography on cellular behavior [16,17]. Each of these methods plays a crucial role in enabling scaffolds to not only support but actively participate in the healing and regeneration process in bone and joint surgery. This review provides a state-of-the-art, comprehensive overview of the functionalization of scaffold-based strategies used in tissue engineering, specifically for bone and joint regeneration. Critical issues and obstacles are highlighted, applications and advances are described, and future directions are identified.

### 1.1. Biological Background

#### 1.1.1. Bone Tissue

**Figure 2:**
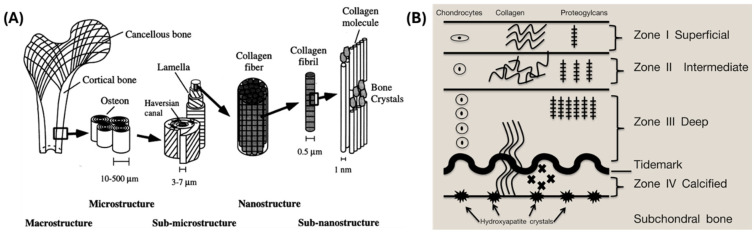
Representative schematics: (**A**) Bone. Image reprinted with permission from Rho et al. [36]. Copyright 1998, Elsevier Ltd. (**B**) Articular cartilage showing the variation in the anatomical structure at the macro-, micron- and sub-micron levels. Reprinted with permission from ref. [37]. Copyright 2016 Elsevier Ltd.

The skeletal system performs various functions, including providing mechanical support, vital organ protection, maintenance of mineral content, hematopoiesis through bone marrow, and locomotion [18,19]. Bones are among the few tissues with regenerative potential, a process that can be impaired in particular circumstances (i.e., critical-size lesions), demanding therapeutic interventions [20]. Bones can be categorized in multiple ways, with one of their classifications based on their composition: cortical or trabecular (Figure 2A). Cortical bone is characterized by its dense and solid structure, which encloses the marrow space, while trabecular bone consists of a honeycomb-like network of trabecular plates and rods dispersed within the bone marrow compartment (both cortical and trabecular bone comprising osteons) [18]. Cortical bone generally exhibits lower metabolic activity compared to trabecular bone, although this characteristic can vary among different species [18].

The predominant cellular components in bone tissue are osteoblasts, osteoclasts, and osteocytes. Activated multinucleated osteoclasts originate from mononuclear precursor cells belonging to the monocyte–macrophage lineage [18,21]. To generate osteoblasts, mesenchymal stem cells (MSCs) must be exposed to the canonical Wnt/β-catenin pathway and associated proteins [18,22]. The Wnt system is also important in chondrogenesis and hematopoiesis; in addition, it may be stimulatory or inhibitory at different stages of osteoblast differentiation [18]. Moreover, osteoclasts are exclusive cells identified with the capacity to resorb bone tissue, while osteoblasts synthesize new bone matrix on bone-forming surfaces [18]. The combined activity of osteoblasts and osteoclasts comprises the bone remodeling process, by which bone is renewed to maintain strength and mineral homeostasis [18]. According to Quarto et al. [20], the successful replacement of bone through tissue engineering relies on the replication of this sequence of events, aiming to replicate and imitate this natural setting by providing cells capable of differentiating into osteoblasts, initiating the release of growth factors, and employing biomaterials to facilitate cellular adhesion, growth, movement, and the deposition of the matrix. Indeed, bone regeneration necessitates an interplay between microenvironmental factors and cells.

#### 1.1.2. Cartilaginous Tissue

Articular cartilage (AC) (Figure 2B) is a compact ECM-rich tissue that deteriorates in response to prolonged mechanical stress. Its innate ability to self-repair is notably limited, especially in aged and osteoarthritic joints, given its avascular nature [23]. Therefore, diffusion serves as a critical mechanism for transporting nutrients and various molecular signals that regulate cell metabolism and maintain the ECM within AC. Understanding the distribution of solutes within cartilaginous tissue is crucial for elucidating its pathologies and developing strategies for ECM repair and regeneration [23,24]. Regarding the cellular component, AC is organized into zones, the outer region of which typically comprises cells that are small in size and exhibit proliferative activity [23]. Furthermore, AC is a hypocellular tissue [25,26,27] with limited progenitor cells [25,28], characterized by low cellular mobility due to its highly pressurized matrix rich in proteoglycans and collagen [25,29].

Given that cartilaginous tissue is avascular, aneural, and alymphatic [25,30], its nutrition occurs through diffusion from the synovial fluid [25,31] and subchondral bone [25,32,33]. In the absence of tissue repair, the newly formed tissue often consists of fibrocartilage, which is biomechanically inferior due to its compositional differences from AC [25]. Concerning tissue engineering as a treatment strategy for cartilage tissue repair, it is understood that the primary objective of this therapeutic modality is to repair or regenerate AC by restoring its structure, architecture, and function [25,34]. Numerous design methodologies, cell origins, biomaterial selections, and fabrication methodologies have been investigated extensively, but there is no consensus in the literature about the best approach yet [25,35]. When compared to autologous and allogeneic grafts, tissue engineering for cartilage restoration presents clear advantages, such as its ability to be tailored to suit the specific needs of each case, thus conforming to the size and configuration of the defect [25].

## 2. Methods of Functionalization

### 2.1. Materials for Scaffold Development

#### 2.1.1. Synthetic Polymers

Synthetic polymers such as PCL, and PLGA offer controlled/predictable degradation kinetics and mechanical properties. These polymers can be used alone or in combination with natural polymers to tailor scaffold properties for specific tissue engineering applications. PLA has been under investigation for nearly five decades as an extensively used bioresorbable polymer [38,39,40]. PLA is produced entirely from renewable sources like corn. In the medical field, it is broadly categorized into two types: L-PLA (predominantly crystalline) and DL-PLA (predominantly amorphous) [41]. In the medical sector, polyglycolic acid (PGA) was initially utilized as biodegradable stitches. It is characterized by strong crystallinity, low solubility, and a high melting point, and derived from glycolic acid through the opening of a cyclic acid di-ester ring [42]. The hydrophilic nature of PGA contributes to its high degradation rate. Typically, after implantation, the implant’s strength decreases to 50%, and within 28 days it further reduces to 90%. Consequently, PLGA, a copolymer of PGA and PLA, finds broader applications in therapeutics. Common biodegradable fibers like Vicryl and polyglactin 910 are composed of PLGA. Hydroxyl–acetic acid, the degradable component of PGA, is either eliminated through the kidneys or metabolized by the liver, ultimately yielding carbon dioxide and water as end products. PGA biomaterials offer a significant advantage due to their non-toxic, aggregative, and biodegradable properties [43,44,45].

The non-hazardous polyester, PCL, is obtained by the ring-opening polymerization of ε-caprolactone monomers, a process that can proceed through anionic, cationic, coordination, or radical polymerization mechanisms [46]. Adjusting its degradation rate by modifying its molecular weight is challenging due to its hydrophobic nature. However, PCL poses a lower risk of degradation owing to its higher crystallinity relative to PGA and PLA. Its excellent strength, good biocompatibility, and biodegradability enable PCL to be formed, molded, extruded, and shaped. These properties make it a commonly used material in drug delivery systems due to its effective medication permeability [42]. PCL, despite its limited biocompatibility, is a versatile biomaterial valued for its rubbery nature, customizable biodegradability, and ability to form various blends, composites, and copolymers. It functions well as a scaffold in tissue engineering and in applications such as surgical sutures and micro- and nano- drug delivery systems [47,48,49]. Park et al. added beta-tricalcium phosphate (β-TCP) to modify PCL-based 3D-printed scaffolds for dental applications [50]. The composite scaffolds, especially those with higher β-TCP content, showed increased surface roughness, porosity, and wettability. These enhancements actively promoted the osteogenic differentiation and proliferation of MSC lines derived from mice [50]. Zehbe et al. prepared PCL foams using a supercritical carbon dioxide foaming technique [51]. This method incorporated the introduction of carbon dioxide into the structure of the polymer and its extraction to form pores. A blend of calcium (Ca) and strontium hydroxides was also employed. The outcome was composite scaffolds of PCL enriched with strontium (Sr)-doped calcium carbonate, having an open structure with a pore size of 100 to 500 μm. The obtained scaffolds exhibited good biocompatibility [51].

Polypropylene fumarate (PPF) is a synthetic, unsaturated linear polyester. Hydroxypropyl fumarate is produced by the reaction of diethyl fumarate with excess propylene glycol in the presence of zinc chloride as an acid catalyst during the initial step of PPF synthesis. Cross-linking of PPF can be achieved through its fumarate double bond. High mechanical strength is attained by properly cross-linking PPF, rendering it highly recommended for bone replacement scaffolds. Furthermore, an osteoconductive surface for bone ingrowth is provided by the porous PPF scaffold [52,53]. Poly(anhydride-co-imides) have been specifically engineered for orthopedic applications, exemplified by polymers like poly-[trimellitylimidoglycinr-co-bis(carboxyphenoxy)hexane)] and poly-pyromellitylimidoalanine-co-1,6-bis(carbophenoxy)hexane]. These polymers demonstrate significantly improved mechanical properties. Their composition involves succinic acid, trimellitylimidoglycine, and trimellitylimidoalanine, yielding compressive strength within the range of 50–60 MPa. Laurencin et al. conducted studies on the mechanical attributes of poly(anhydride-co-imides) as scaffolds for bone tissue engineering applications. An assessment of the osteo-compatibility of these polymer materials was performed using the rat tibial model. The findings revealed that untreated imperfections healed within 12 days. Conversely, imperfections treated with poly(anhydride-co-imides) facilitated endosteal bone growth by the 3rd day and formed cortical bone bridges around the implanted matrices by the 30th day indicating the matrices’ osteo-compatibility [54,55]. Polyurethane (PU) stands as a remarkably versatile and cost-effective material widely employed across numerous medical applications. Its attributes encompass stiffness, flexibility, mechanical robustness, and elasticity. Yet, its most crucial characteristic lies in its ability to replicate specific biological structures within the body, notably resembling bone. Additionally, PU can be engineered to be entirely biocompatible, suitable for permanent implantation, and can also be designed to be biodegradable, serving as resorbable scaffolds to aid in tissue regeneration [56].

#### 2.1.2. Natural Polymers

As natural polymers, collagen (COL) and gelatin have been considered excellent materials for tissue engineering applications in terms of their biocompatibility [57,58]. COL, the main constituent of cartilage and bone, is mixed with other polymers or bioceramics to modify its properties [59]. Even though its degradation can be mitigated by cross-linking, its self-assembling nature is problematic for the fabrication of scaffolds [60]. Gelatin is biocompatible [61] and promotes cellular functions [62] as well as the ECM [63]. However, gelatin demonstrates poor printability and mechanical properties [61,64]. Regardless, gelatin continues to be frequently used in tissue engineering due to its low cost and easy preparation [63,64,65,66]. Mixing gelatin with HA can increase its viscosity for 3D printing [62]. Gelatin can also be cross-linked with methacrylamide or methacrylate [62,67,68,69], improving its mechanical properties and making it suitable for use in MZSs (multizonal scaffolds) for cartilage and bone regeneration. Gelatin can be used to release growth factors for establishing an anti-inflammatory environment and for the generation of tissues in MZSs [65,68,69,70].

Natural silk fibroin (SF) is one of the most common biocompatible polymer components used in textiles and the most widely used substance for cartilage tissue engineering. It has a range of advantages, including biocompatibility, unique morphological structures, and adjustable mechanical and porous properties. SF-based scaffolds offer controllable porosity and tunable mechanical properties; they are also very suitable for a wide range of applications [71,72]. However, SF hydrogels alone have poor mechanical properties [73,74], even after cross-linking. To address this limitation, SF is often combined with other materials, such as chitosan [75], peroxidase [71], or chondroitin sulfate (CS) [76]. These composite scaffolds exhibit improved mechanical properties, with compressive moduli ranging from 350 KPa to 6.7 MPa. Additionally, SF-based tri-layered multizonal scaffolds have shown excellent in vitro MSC adhesion, proliferation, migration, and differentiation, as well as in vivo neocartilage tissue formation and high expression of type II COL [77].

Chitosan, derived from chitin found in shrimp shells, is highly biocompatible, osteoconductive, and osteoinductive. It degrades slowly due to its high degree of deacetylation and promotes cell differentiation, making it suitable for constructing both cartilage and bone layers in MZSs. In the bone layer, chitosan facilitates the growth of calcium phosphate (CaP) crystals and osteogenesis markers, promoting osteogenic cell proliferation and adhesion. In the cartilage layer, chitosan promotes chondrogenic differentiation and enhances glycosaminoglycan (GAG) production. Chitosan-based MZSs exhibit a high compression modulus and can be enhanced by mixing with proteins like SF, gelatin, or COL, or by forming polyelectrolyte complexes with other polysaccharides. The degradation rate of chitosan-based MZSs can be tailored by adjusting scaffold compositions, and cross-linking techniques can further reduce the degradation rate, although they may lead to cell toxicity and reduced cartilage matrix production.

HA, a major component of cartilage tissue, supports chondrogenesis and is advantageous for MZS fabrication, particularly in the cartilage layer. HA offers good biocompatibility, an appropriate degradation rate, and high printability. In bilayer scaffolds, HA hydrogels promote the differentiation of MSCs into chondrocytes in the cartilage layer and into osteoblasts in the bone layer. HA can be combined with other polymers to enhance its properties, such as mixing with chitosan to enhance cell differentiation or with PCL to increase the production of cartilage matrix components like type II COL and GAGs. Mixing HA with polyglycerol polymers can tailor its chondrogenicity, although the compressive properties of the resulting scaffold may remain low.

#### 2.1.3. Bioceramics

In recent decades, bioceramics have been commonly employed in the restoration and replacement of damaged tissues due to their numerous advantages, notably their precise chemical composition, crucial for the integration of both hard and soft tissues [78,79,80]. One of the major components of human bone, about 65% of total bone mass, is an inorganic solid, namely carbonate HAp [18]. These carbonate HAp crystals are recognized for their growth within the COL I networks between cells, aiding in bone ingrowth and preventing osteolysis [81]. Cox et al. developed a systematic 3D printing method to achieve customized macropore interconnectivity in HAp-polyvinyl alcohol (PVOH) scaffolds [82]. Key factors such as bulk interconnection, set at 500 µm macropore sizes, and surface roughness (resulting from HAp: PVOH mixture) played a crucial role in promoting osteo-conduction. These factors aided in scaffold integration within the body, enabling osteocyte migration and vascularization and contributing to the bone formation process [82]. While various composites involving HAp have expanded, HAp remains a predominant biomaterial. These include combinations like HAp–chitosan, HAp–fibronectin, HAp-PCL, and HAp-COL, aimed at improving mechanical strength, promoting osteoinduction, enhancing vascularization, and encouraging localized chondrogenic differentiation. Particularly noteworthy is the imminent clinical application of a specific variant of HAp [83,84,85,86,87]. Woeszn et al. produced microporous HAp scaffolds with 450 µm pore sizes using a combination of stereolithography and ceramic gel casting [88]. They utilized a photosensitive liquid resin containing a water-based thermosetting slurry within the mold. The mold resin and sintering process were utilized to attain the desired characteristics. Subsequently, the finalized scaffolds were cultivated with MC3T3-E1 cells for a 14-day period, promoting deep cell penetration and exceptional osteogenesis [88].

Over a period spanning twenty years, β-TCP scaffolds have achieved substantial recognition within clinical contexts, serving as effective substitutes for bone grafts across numerous orthopedic applications [89,90,91,92,93,94,95]. β-TCP exhibits multiple sites capable of accommodating ionic substitution, allowing for the inclusion of diverse elements into its foundational ceramic structure [96]. Additionally, research indicates that ceramic materials may inherently possess antibacterial properties, offering the potential to mitigate or reduce bacterial adhesion—an issue frequently encountered with implantable devices. Incorporating materials like copper (Cu), zinc (Zn), fluoride (F^−^), iron (Fe), magnesium (Mg), and/or silver (Ag) within a ceramic matrix could further enhance their properties, potentially mimicking composites with uniformly dispersed metal particles and theoretically achieving isotropic characteristics [97,98,99,100,101,102,103,104,105].

Bioactive glass consists of a silicone oxide network and a specific network-modifying oxide, like calcium oxide, magnesium oxide, or potassium oxide. The connection between oxygen and silicon on both sides is referred to as a bridging bond, while substituting silicon on one side with another network modifier forms a non-bridging bond. Like inert ceramics, bioglass ceramics containing 60% or more silica exhibit similar behaviors. Bioactive glasses containing less than 10% Na_2_O and without K_2_O and Al_2_O_3_ show promise in encouraging apatite deposition and facilitating bone regeneration [106]. Nommeots-Nomn et al. utilized robocasting to create bioglass scaffolds featuring 150 µm interconnected pore sizes (41–43% porosity) with compressive strengths measuring between 32 and 48 MPa. These scaffolds demonstrated network connectivity (NC) akin to the 45S5 bioglass. The process involved using ICIE16 and PSrBG compositions containing <50 molecular percentage SiO_2_ to maintain an amorphous structure and achieve NC comparable to 45S5 bioglass. The manufactured scaffolds were compared to bioglass compositions ranging from 13 to 93 volume percentage, revealing that the 3D porous scaffolds exhibited similar NC values to 45S5 bioglass using lower silica contents. Furthermore, the Pluronic F-127 binder proved effective as a universal binder for bioactive glasses regardless of their composition and reactivity. The results indicated that scaffolds based on ICIE16 and PSrBG were highly reactive and notably accelerated bone regeneration [107]. 3D-printed mesoporous bioactive glass (MBG) scaffolds also display remarkable capacity for apatite mineralization and sustained drug delivery when appropriately adjusted. Their notably high mechanical strength, approximately 200 times greater than conventional PU foam templated-MBG scaffolds, underscores their potential suitability for applications demanding high compressive strength, indicating the potential for clinical translation [108].

Over recent decades, calcium phosphate cements (CPCs) have been utilized in both in vitro and in vivo studies to demonstrate their potential for forthcoming orthopedic treatments. These applications include the mending of fractures and addressing defects, showcasing their promise for future clinical use in orthopedics [109,110,111,112,113,114]. The characteristic of CPCs setting at low temperatures further enhances their utility in orthopedic bioengineering. This feature serves as an ideal foundation for drug loading and delivery, amplifying their usefulness in orthopedics [115]. Aside from their accelerated hardening, CPCs are recognized for their exceptional biocompatibility and bioactivity, despite limitations in controlling their resorption rate [116]. Gbureck et al. explored the use of 3D-printed CPC scaffolds, enhancing their hardening capabilities through immersion in a diluted phosphoric acid solution. Additionally, their research demonstrated the potential of CPCs to accommodate bioactive agents by crafting bioceramic implants via precise printing methods. These implants were designed to include angiogenic factors, aiming to advance tissue healing processes [117,118].

#### 2.1.4. Metals

The intricate and diverse porous structure of bone encompasses a range of pore sizes spanning from the macro- to the nano- scale. Consequently, 3D-printed implants aim to replicate these porous structures to closely resemble natural bone [119]. Unlike conventional solid implants fabricated from materials like titanium (Ti), stainless steel, or cobalt–chromium, the incorporation of macro- or micropores in 3D-printed implants offers space for the growth of cells, tissues, blood vessels, and nerves, facilitating biological integration [120]. The prevalent raw materials utilized for 3D-printed implants predominantly consist of Ti and Ti-based powders. While these materials demonstrate commendable biocompatibility and osteoconductive properties, their susceptibility to pit corrosion when subjected to fluidic environments under load-bearing circumstances can lead to potential implant failure. Moreover, the disparity in stiffness and elastic modulus between Ti-based implants and natural bone presents an ongoing challenge that necessitates resolution. Through integration with 3D printing methodologies, an alloy such as Ti–24Nb–4Zr–8Sn comprising a high strength (~800 MPa) and elastic modulus (49 GPa), experienced a notable reduction in modulus to 4.36 GPa. This value closely approximates the modulus of cancellous or trabecular bone [121]. Palmquist et al. investigated the osseointegration of solid Ti6Al4V implants alongside EBM-printed porous implants, presented in disk and cylindrical shapes [122]. These implants were bilaterally inserted in the subcutaneous region of the femur dorsum in sheep. Their study revealed successful osseointegration for both solid and porous implants following a 26-week implantation period. Notably, the porous implants exhibited an increased bone contact rate of up to 57%, the highest among all porous implant variations [122].

Tantalum (Ta) has been widely favored in orthopedic and dental applications since the 1940s, notably for vascular clips, cranial defect repair, nerve repair, and bone markers, owing to its favorable chemical stability and exceptional biocompatibility [123]. However, its extensive use was historically limited due to the substantial production expenses and the complexities linked to creating modular implants [124]. Clinical studies on porous Ta implants, like those for spinal, tibial, and acetabular applications, have showcased their effectiveness across diverse clinical scenarios [125]. Advancements in manufacturing techniques such as laser-engineered net shaping (LENS), selective laser melting (SLM), and spark plasma sintering have been employed to create both porous and solid Ta components [126].

It is important to note that materials with Young’s moduli higher than human bone typically cause stress shielding, potentially leading to fractures, loosening, and bone weakening [127]. To address this, introducing porosity to materials could promote better and long-lasting stability for effective fixation [128]. Biodegradable metals, such as Mg-, Ca-, Fe-, and Zn-based alloys, exhibit bioactive traits and can fully degrade within the human body. These metallic implants offer both biological functionality and mechanical reinforcement. Their low Young’s moduli, like bone, provide an advantage in minimizing the negative impacts of stress shielding.

Zn has also been regarded as a promising biodegradable metal for orthopedic implants due to its degradation rate, which falls between that of Fe and Mg [129]. Scientists have discovered that Zn possesses strong anti-atherogenic properties [130]. However, with a small difference between its low melting and boiling points and its high vapor pressure, it is challenging and dangerous to fabricate porous Zn-based implants with 3D printing techniques [129]. Qin et al. devised an enhanced gas flow mechanism to counteract the adverse impact of Zn metal evaporation during the laser powder bed fusion process, as determined by numerical analysis [130]. Through fine-tuning the shielding flow and laser energy input, pure Zn implants were successfully fabricated with a density exceeding 99.90% [130]. Utilizing an AM technique, the incorporation of rare earth cerium into Zn has notably enhanced the ultimate tensile strength and resistance to creep [131]. Meanwhile, Yang et al. opted for rare earth lanthanum (La) as a compatible interface layer between carbon nanofiber (CNF) and the Zn matrix. This addition of La substantially bolstered both the tensile strength and ductility of the CNF–La–Zn composites, with La also improving the anti-tumor performance of these composites [132].

Fe-based alloys possess ample strength for application in bone implants; however, it is crucial to augment their corrosion rate within the body to synchronize with the natural healing rate of bone. Chou et al. employed inkjet 3D printing to synthesize of iron-manganese alloy-based (Fe–Mn) scaffolds, showcasing mechanical properties akin to natural bone, featuring 36.3% open porosity. These scaffolds exhibited favorable corrosion rates compared to pure Fe, permitting cell infiltration through the open pores [133]. Surface modifications and topological design adjustments on 3D-printed Fe scaffolds were also suggested to enhance osteogenesis properties [134,135,136]. Zhang et al. integrated magnetic Fe_3_O_4_ nanoparticles, PCL, and bioactive glass into composite scaffolds using a 3D Bio-plotter [137]. These scaffolds displayed a compressive strength ranging from 13 to 16 MPa, with an evenly distributed 60% porosity within the structure. Incorporating magnetic Fe_3_O_4_ nanoparticles provided the scaffold with a magneto-thermal effect, enhancing its cellular biological functions. Moreover, the scaffold was loaded with doxorubicin hydrochloride, an anti-cancer drug to enhance the therapeutic effect and maintain a steady drug release [137].

#### 2.1.5. Xenografts

Graft materials can be classified according to their source. Autogenous bone grafts, which are transferred from the same individual who will receive the graft, are the gold standard. Allografts are transferred from another individual of the same species, such as cadavers. Xenografts are transferred between different species, for example, bovine or equine grafts. Finally, alloplasts encompass bone grafts produced with synthetic materials, as described in previous sections, with the addition of growth factors, such as bone morphogenetic protein or platelet-derived growth factor, and/or cells [138].

Until recently, xenogeneic grafts were avoided in reconstructive surgeries of the foot and ankle due to concerns about their antigenicity and possible rejections [138]. Each constituent stage of the tissue graft development cycle can lead to alterations in its mechanical, chemical, and even biological properties, and these alterations can define the success or failure of the implant [139,140,141,142]. However, with the advent of new processing and sterilization techniques, there has been a notable decrease in cases of immunogenicity, allowing for a renewed emergence in the use of xenografts [138]. Currently, there are several commercially available xenografts, such as Artegraft^®^ (LeMaitre Vascular, Inc., Burlington, MA, USA), Bio-Oss^®^ (Geistlich Pharma AG, Wolhusen, Switzerland), CardioCel^®^, (LeMaitre Vascular, Inc., Burlington, MA, USA), among many others [139]. As an advantage, xenografts can offer structural reinforcement and osteoconduction; however, osteoinduction and osteogenesis are still unachievable [138]. Patient-related factors concerning the utilization of xenogeneic scaffolds should also be considered beforehand, knowing that specific cultural and faith-based communities might object to animal tissue utilization in any capacity [142,143].

### 2.2. Advanced Technologies for Functional Scaffold Engineering: 3D Printing

Over the last decade, the engineering of bone and joint scaffolds has advanced to fulfill biological and structural functions predominantly in preclinical settings, given that the microstructure of trabecular bone has not been adequately replicated for clinical applications [144]. One of the fundamental issues that prohibit the accurate replication of trabecular bone microstructure in scaffolds is the nonuniformity in different directions of measurement that varies significantly throughout bone trabecula, otherwise known as anisotropy [145,146]. Furthermore, the different processing parameters of scaffolds, including meshing, image resolution, sample boundary conditions, support material type, among others, have been shown to significantly affect mechanical strength and stiffness as well as reproducibility of the properties of the scaffold [147]. An optimal balance of permeability for cell absorption, proliferation, and bone ingrowth with mechanical stress has yet to be achieved in vivo [146,148].

In bone tissue engineering specifically, mechanical strength is of key consideration due to the load-bearing requirements of various bone defects. Scaffolds fabricated for bone tissue engineering should have comparable strength to the native bone tissue. However, material selection with varying inherent mechanical strength can be optimized to allow for increased porosity [149]. Different fabrication techniques vary in complexity and application but share the common goal of enhancing the scaffold’s interaction with native tissues. For example, electrospinning is a versatile and widely used technique in the fabrication of scaffolds, known for its ability to create fibers with diameters ranging from nanometers to micrometers. This process involves applying a high voltage to a polymer solution, which results in the formation of fine jets that solidify into fibers as they are collected on a grounded target [150]. Electrospinning parameters like voltage, flow rate, and solution concentration can be adjusted to control fiber diameter, where smaller fiber diameters increase surface area-to-volume ratios, enhancing cellular attachment and proliferation [151]. In addition to cellular attachment, a high area-to-volume ratio allows for the incorporation of growth factors, drugs, or bioactive nanoparticles into the fibers during fabrication. Furthermore, the microarchitecture created by electrospinning includes high porosity and a tunable fiber alignment. Aligned fibers can direct the orientation and elongation of cells, promoting organized tissue formation and improving mechanical properties [152]. For tissue-engineered scaffolds, mean pore size is critical because it influences cell migration and attachment. Conflicting reports on the ideal pore size result from the unclear relationship between scaffold pore size and cell activity [153]. The growing use of bone scaffolds requires high performance, as their porous structures can be tailored to repair damaged bone tissues. Traditional manufacturing methods limit the development of porous scaffold structures, but AM technology has made this process relatively easier [154].

**Figure 3:**
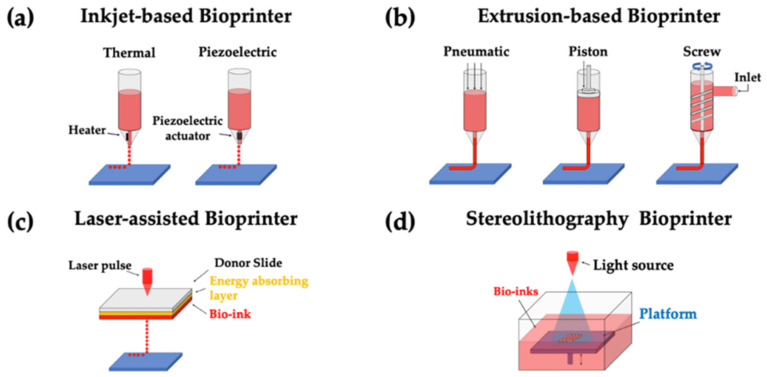
Visualization of various bioprinting techniques. Reprinted from ref. [159].

In terms of fabrication techniques, AM or three-dimensional (3D) printing offers an efficient approach to creating customized functional scaffolds that enable the controlled release of bioactive molecules and cells for tissue regeneration [1,155]. Living cells can be incorporated within the fibers during the fabrication process or seeded onto the matrix afterward for colonization. The capability to adjust 3D-printed scaffolds to attain ideal biomechanical characteristics and simulate the natural ECM has been shown to enhance the effectiveness of their implantation [1,156,157,158]. Conventional technologies for 3D scaffold engineering include stereolithography, fused deposition modeling, selective laser sintering, 3D bioprinting, inkjet-based bioprinting, extrusion-based bioprinting, laser-assisted bioprinting, and 4D bioprinting (some examples shown in Figure 3) [144]. The below sections provide a brief overview of the techniques, their advantages and disadvantages, and advances in bone and joint engineering that have made use of these techniques.

#### 2.2.1. Stereolithography

Stereolithography uses the polymerization of liquid-based resins to create three-dimensional scaffolds layer-by-layer. Lasers or UV light are used as sources of irradiation [160]. Polymerization of the scaffold is heavily dependent on resin properties and penetration of irradiation. Commonly used resins that have been employed with the stereolithography technique include polyacrylate and epoxy macromers, which are low-molecular-weight multi-functional monomers or macromers that form cross-linked bonds with glassy, rigid macroscopic properties, less than ideal for bone and joint scaffolds. Polymer–ceramic slurries using materials such as β-TCP have also been employed [161,162]. The advantages of stereolithography 3D printing include the rapid curing of high-viscosity materials, thereby maintaining the controllability of the printing process and allowing the creation of high-accuracy and high-resolution microstructures [161].

Many disadvantages of stereolithography exist, however, including the materials that can be used as well as the process itself. The resins selected for stereolithography must be photosensitive, which limits the number of potential materials. Furthermore, only one resin can be used at a time during the printing process. Many of these resins have been shown to have poor biocompatibility and possible cytotoxicity [161]. For example, acrylic polymers have been shown to exhibit cytotoxicity in vitro. Methods such as further autoclaving, passivation with polydimethylsiloxane (PDMS) coating, and Soxhlet extraction have been proposed to reduce cytotoxicity, although some may affect mechanical performance [163]. Other photo-polymerizable resins include PEG, PCL, poly(D,L-lactide) (PDLLA), and PPF [164]. As for the printing process itself, over-curing and under-curing of the resins affects the mechanical properties of the finished product. Residual resin must also be removed so as not to interfere with the intended scaffold structure. Printing of more complex structures and designs requires complex polymerization steps and rinsing with each layer, thereby requiring more time or more advanced equipment [161].

#### 2.2.2. Selective Laser Sintering

Selective laser sintering uses a guided laser beam as an energy source to partially melt powder, allowing for binding between particles. This results in a solid final product with porous properties. Its advantages include the high speed and low cost of the process as well as the ability to fabricate porous microstructures which can mimic trabecular bone. The disadvantages include the lack of precision in printing relative to stereolithography. Furthermore, crystalline polymers tend to shrink and morph the final product. Materials are limited to those with a crystallization temperature below the melting point, including polyamide (PA), polystyrene (PS), thermoplastic elastomers (TPE), polypropylene (PP), and semi-crystalline thermoplastics [165]. More recently, SLM has been used to create load-bearing metallic scaffolds from metal alloys, which are then filled with ceramic materials, such as zirconium dioxide, titanium dioxide, HAp, and wollastonite, with spark plasma sintering. This method has the advantage of creating lightweight yet mechanically robust structures [166].

#### 2.2.3. Inkjet-Based Bioprinting

Inkjet-based bioprinting uses bioinks, which are composed of cell solutions. The printer is used as either a thermal or piezoelectric actuator to generate droplets, which are forced, either continuously or on demand, by a nozzle onto the substrate [167]. Advantages of this method include unlimited design possibilities due to the ability to print nonuniform shapes and high spatial resolution. However, its limitations include cell aggregation during the drying process, which reduces the final product’s quality, as well as decreased structural stability with low-concentration solutions [168].

#### 2.2.4. Extrusion-Based Bioprinting

Extrusion-based bioprinting uses pneumatic, piston-, or screw-driven forces to extrude a biomaterial or bioink out of the printer head and onto the desired surface [169]. The advantages of extrusion-based bioprinting include the ability to print high solution concentrations at a rapid rate, resulting in high structural integrity. The limitations of bioprinting include the number of bioinks available and the fact that the pressure differentials and shear generated while printing can result in cell death [168,170].

### 2.3. Microarchitectural Design and Surface Topography

Microarchitectural designs and surface topography are critical components in the functionalization of scaffolds. These modifications involve engineering the scaffold’s structure at the micro- and nanoscopic levels to more closely mimic the surrounding native tissue. Surface topography plays a crucial role in enhancing the performance of scaffolds by improving cell adhesion and proliferation through mechanical cues physically transduced to cells [171]. In addition to specific biochemical cues, these physical cues are more general regulators of cellular behavior and function [172]. By improving the surface physicochemical properties of scaffolds, cells are better able to adhere and begin their regenerative process. There are various techniques for improving topography; common examples include acid etching and plasma treatment. For example, plasma treatment, where inorganic and organic compounds are delivered onto implant surfaces via plasma jet deposition, has been shown to improve hydrophilicity and, thereby, cellular adhesion [173,174]. Conversely, acid etching involves immersing implants in acidic solutions, creating small pits and increased surface roughness for osteoblastic adhesion [17]. On the other hand, microarchitectural features that influence performance include pore size and shape, porosity, surface area-to-volume ratio, and mechanical strength.

#### 2.3.1. Pore Size and Porosity

Porosity is an important criteria for evaluating porous structures, as it affects nutrient transport, cell adhesion, proliferation, and bone formation, especially in 3D-printed scaffolds for bone regeneration [144,154]. Pore size has also been shown to influence cell migration and proliferation within the scaffold, in addition to nutrient diffusion and waste removal. In bone tissue engineering, the optimal pore size for osteoblast activity within scaffolds is still debated, as there have been conflicting reports [175,176,177]. Generally, scaffolds with pore sizes of 20–1500 μm have been used [153,178]. In terms of angiogenesis, the minimum porosity necessary for blood vessel infiltration and regeneration is approximately 30–40 μm [179,180]. However, studies have demonstrated improved angiogenesis in nondegradable scaffolds at pore sizes of 160–300 μm [181,182]. Additionally, pore size was shown to improve mineralization and improve mechanical properties of the tissue engineering scaffolds. For example, Karakeçili et al. used chitosan, COL type I, and nano-HAp to prepare biomimetic porous cross-linked scaffolds to evaluate its effects on scaffold characteristics and osteoblast differentiation [183]. Pore sizes between 70 and 250 μm were indicated to have contributed positively to the mineralization process.

**Figure 4:**
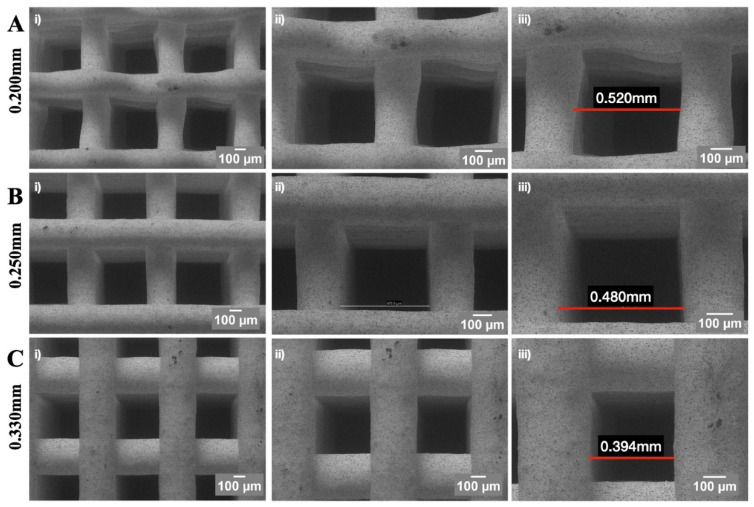
Scanning electron micrographs of scaffolds with different pore dimensions and porosities (**A**–**C**); at different magnifications (**i**–**iii**). Reprinted from ref. [189].

On a similar note, percent porosity in scaffold architectures also remains important, as it promotes nutrient flow and facilitates an open structure for cells to occupy and differentiate, as opposed to a closed structure that could act as a barrier to the survival of cells [184]. The optimal porosity range is important for successful scaffold applications as it also helps mimic the natural porous structure of bone while allowing for improved vascularization and osteoconduction [185]. A porosity between 70 and 90% has been associated with superior bone regeneration outcomes [186]. For example, Takahashi et al. found that thicker (22–42 μm) polyethylene terephthalate (PET) fibers had more MSCs attached, and that the size, surface area, and porosity of the non-woven fabrics affected MSC attachment, proliferation, and differentiation, providing valuable information for the design of tissue engineering scaffolds [187]. A study by Wei et al. tested the biocompatibility of porous Ta in vitro and in vivo [188]. The porous Ta coating promoted adhesion, aggregation, and cell proliferation, while in vivo studies in a canine model showed that defects treated with a high-porosity Ta coating (70–85%) produced positive healing outcomes with adequate osteointegration between newly regenerated bone and the implanted tissue engineering device [188]. Corroborating these findings, Arbex et al. compared the effects of porosity and surface area on cell proliferation in vitro using 3D-printed β-TCP scaffolds, indicating improved cellular proliferation in scaffolds with higher porosities (Figure 4) [189]. However, there is a lack of consensus in the literature pertaining to the optimal pore size and/or porosity of tissue engineering devices due to different processing conditions, materials, and scaffold manufacturing techniques utilized [154]. Nonetheless, it is important to note that pore size and porosity can also have an effect on the mechanical properties of a scaffold [190]. Although higher porosity and pore sizes may facilitate osteogenesis and angiogenesis through nutrient delivery, higher porosities can compromise the mechanical strength of the implanted device [190].

#### 2.3.2. Pore Configuration

**Figure 5:**
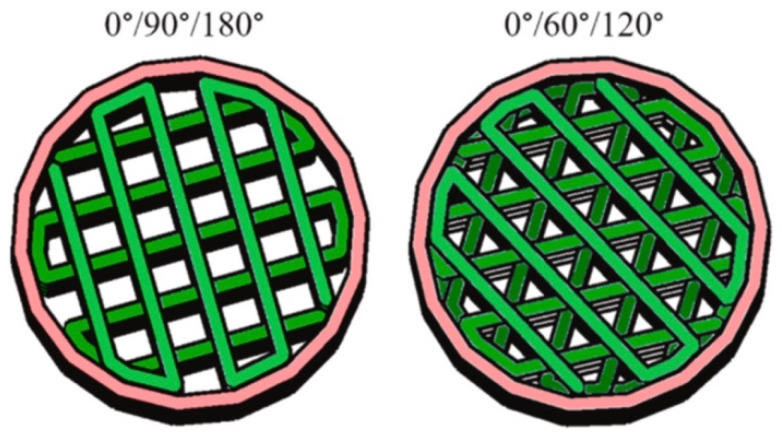
Examples of different scaffold pore configurations/raster patterns achievable through 3D printing. Reprinted with permission from ref. [192]. Copyright 2021 Elsevier Ltd.

Scaffolds or grafts have traditionally been static physical structures that attempt to mimic the complex dynamic behavior of in vivo microenvironments [191]. Furthermore, these devices provide a minimal opportunity to mimic the critical structural remodeling and biomechanical signaling that extracellular matrices (ECMs) demonstrate during normal tissue development. The current approaches to addressing these limitations rely on cellular modification, such as the cellular deposition of ECM proteins within a scaffold [191]. While they have been established to be a viable technique, they can be difficult to apply in situations where biochemical composition is of paramount importance. Scaffolds with interconnected lattice structures can be achieved in a multitude of ways. With the end goal of preserving the porous nature of the constructs, it is imperative that gaps between the individual printed lines (rods or struts) that make up a layer exist. These rods in a given layer, typically 3D-printed at an angle or perpendicular to each other in a periodic or aperiodic fashion, commonly referred to as raster patterns (Figure 5), have been found to greatly influence cell growth [192]. An example of a study that analyzed different raster orientations of 0°/90°/180° and 0°/60°/120° reported differences in the mechanical properties of the scaffolds [193]. This can be interpreted by closely observing pore shapes—square-shaped structures exhibit low shear stress, while triangular structures typically are stressed to a higher extent due to large stress concentrations at the vertices. Ostrowska et al. also performed similar studies and discovered that some orientations perform better in vitro due to the tortuous nature of the struts (especially orientations of 0°/15°/30°), which helped retain the majority of the cells within the scaffold architecture [192,194]. Hence, it can be concluded that while material properties (including microgeometry) influence various properties of scaffolds for biomedical applications, microgeometry could facilitate the chances of their success in vitro or in vivo.

### 2.4. Bioactive Molecules

The integration of bioactive molecules into scaffolds is a fundamental approach to enhancing their biological functionality and therapeutic efficacy in bone tissue engineering applications. Bioactive molecules, including but not limited to growth factors, peptides, proteins, and pharmacological agents, are essential regulators of cellular processes, which are vital to tissue regeneration and repair. In order to achieve the best therapeutic outcome, functional scaffolds must release their bioactive molecules in a time-coordinated and sequential manner that best mimics physiological patterns [195]. Various loading techniques have been developed to provide different release strategies for different defect types.

During the fabrication of surface-functionalized implants, bioactive molecules are introduced to the surface of biomaterials via physical absorption, chemical conjugation, or ligand–receptor binding. Physical adsorption is the simplest method, involving the direct application of bioactive molecules onto the implant surface through techniques like dipping, spray coating, or drop casting [196]. This approach is straightforward and allows for easy loading of various molecules. However, it often results in a burst release pattern, where a large portion of the bioactive agents is rapidly released shortly after implantation [197]. While providing an immediate therapeutic effect, this method may not be suitable for long-term regeneration purposes [196]. On the other hand, chemical conjugation involves covalently binding bioactive molecules to the implant surface, typically through the use of cross-linking agents or surface activation techniques [198]. This method offers more stable attachment and can provide a more sustained release of bioactive agents compared to physical adsorption. Chemical conjugation allows for better control over release kinetics and can potentially maintain therapeutic concentrations at the target site for extended periods [199]. However, this process can be more complex and bioactive molecules, especially peptides, are easily lost through degradation in vivo [200]. Lastly, ligand–receptor binding utilizes specific molecular interactions to attach bioactive agents to the implant surface. This approach often involves modifying the implant surface with receptor molecules that can selectively bind to specific ligands or bioactive agents [201]. Ligand–receptor binding can offer the highly specific and controlled attachment of bioactive molecules, potentially allowing for more precise targeting and controlled release. However, this method may be limited by the availability of suitable ligand–receptor pairs and the complexity of surface modification [198]. As such, more advanced techniques like sustained release by encapsulation, preprogrammed release, and stimuli-responsive release have also been explored.

The sustained release of biomolecules using encapsulation is a widely employed strategy in drug delivery and tissue engineering applications. This approach involves entrapping bioactive molecules within a protective matrix or carrier system. The encapsulation process can be achieved through various methods, including physical entrapment in hydrogels, covalent conjugation to polymer networks, or encapsulation within micro- or nanoparticles [202,203]. The primary goal of sustained release is to maintain therapeutic concentrations of the biomolecules at the target site over an extended period, thereby enhancing their efficacy and reducing the need for frequent administration. The release kinetics can be tailored by manipulating the properties of the encapsulating material, such as its degradation rate, porosity, or responsiveness to environmental stimuli [204]. Furthermore, this encapsulation protects polymer bioactive molecules from degradation by proteolysis upon implantation [205].

Preprogrammed release patterns allow for precise control over the timing and dosage of bioactive molecule delivery. This approach often utilizes advanced scaffold designs incorporating multiple compartments or layers, each programmed to release specific biomolecules at predetermined intervals [206]. For example, a scaffold might be designed to release an initial burst of anti-inflammatory agents, followed by a sustained release of growth factors to promote bone formation, and finally, a delayed release of angiogenic factors to enhance vascularization. This sequential release can mimic the complex signaling cascades involved in natural bone healing processes, potentially enhancing regenerative outcomes [205,206].

Finally, stimuli-responsive release systems respond to specific environmental cues to trigger the discharge of bioactive molecules [207]. Commonly used stimuli include pH and/or temperature changes; enzymes; magnetic fields; and mechanical factors. While this delivery system has seen success in systemic treatments, including cancer, it has seen limited success in bone tissue engineering applications [208]. Bone regeneration requires long-term drug release, where the stimuli-responsive system is limited by its short duration of action and irreversible responsive release [208]. Nevertheless, this approach offers the potential to reduce side effects and improve therapeutic efficacy by delivering bioactive agents at precisely desired locations and time points. Each of the methods for loading bioactive molecules offers the potential for more sophisticated and tailored delivery of bioactive molecules, potentially leading to improved outcomes in bone and joint tissue regeneration.

Among the various growth factors/bioactive molecules, vascular endothelial growth factor (VEGF) is recognized for its high angiogenic potential and can be expressed by various cells in the human body, whether endothelial or non-endothelial, including tumor cells [209]. It is also known that VEGF exerts a significant influence on bone repair [210] and that during endochondral bone formation, the factor plays a role in recruiting osteochondroprogenitor cells, inducing the formation and subsequent resorption of cartilage for its replacement with bone tissue [210,211,212]. Nevertheless, when VEGF is inhibited, bone regeneration is compromised as it disrupts the transformation of the cartilaginous callus into a bony callus [210,213]. Moreover, the existing literature already reports that the exogenous administration of VEGF enhances the formation of mineralized bone in bone defects [210,213,214,215], and also contributes to the regulation of osteoclast maturation and differentiation, playing a crucial role in bone remodeling [210,213,216,217].

Although the literature highlights the considerable potential of VEGF to facilitate bone formation through various mechanisms, the therapeutic delivery of VEGF remains largely in the preclinical phase [210]. The ideal dosage, treatment duration, and delivery methods for VEGF-loaded scaffolds are still being investigated, which delays their progression to clinical trials [210]. Scaffolds that can release VEGF gradually in physiologically appropriate quantities are required, and careful consideration is necessary when applying exogenous VEGF, especially in the absence of evidence indicating VEGF reduction in patients [210]. In a study conducted by Casarrubios et al., the team fabricated macroporous scaffolds using silicon-substituted HAp with both nanocrystalline and crystalline microstructures and functionalized them with VEGF using simple impregnation methods [218]. Based on observations, the authors reported that the presence of the respective factor on the scaffold’s surface increased endothelial cell proliferation [218]. Regarding the microstructure, it was noted that cases of crystalline microstructure favored the in vitro proliferation and differentiation of pre-osteoblasts, showing superior outcomes regarding the volume of newly formed bone, trabecular thickness, and vascularization of the implant [218]. In contrast, nanocrystalline microstructure scaffolds exhibited detrimental effects on bone defect treatment in vitro, leading to decreased bone ingrowth, thinner trabeculae, reduced osteoblast presence, and increased osteoclast presence [218].

Continuing to explore ideal methods for the gradual release of physiologically appropriate levels of VEGF, Zha et al. [219] suggested the potential use of progenitor cell-derived exosomes enriched with VEGF. Exosomes, ranging from 50 to 200 nm in size, are nano-sized extracellular vesicles composed of a complex mixture of proteins, nucleic acids, and lipids, which are promising therapeutic nanoparticles for disease treatment [219]. Moreover, owing to their exceptional biocompatibility and efficient cellular internalization, exosomes exhibit significant potential as optimal drug or gene delivery carriers in regenerative medicine [219]. In their investigation, Zha et al. [219] showed that engineered exosomes fulfill two key roles: firstly, acting as an osteogenic matrix to trigger MSC differentiation towards bone formation, and secondly, serving as a gene carrier for controlled release of the VEGF gene to enhance vascular remodeling [219]. In vivo experiments also confirmed the effectiveness of exosome-modified bone scaffolds in promoting the regeneration of vascularized bone tissue [219]. Basic fibroblast growth factor and bone morphogenic protein-2 are two growth factors that can be integrated into scaffolds for delivery to bone defects. Basic fibroblast growth factor facilitates wound healing through the recruitment of cytokines and induction of angiogenesis. Bone morphogenic protein-2 induces bone formation through MSC differentiation into osteoblasts. Ding et al. found that when basic fibroblast growth factor and bone morphogenic protein-2 were incorporated into a fibrous scaffold, vascularization and immunomodulatory effects, such as macrophage polarization and the differentiation of periodontal ligament cells, were enhanced in the healing of periodontal bone defects [220].

Like growth factors, microRNAs have been implemented in a preclinical setting to induce bone tissue regeneration. Micro and anti-microRNAs have been shown to induce the expression of runt-related transcription factor 2, which in turn induces osteogenesis [221]. MicroRNAs also play a role in angiogenesis, which stimulates osteogenesis and further increases angiogenesis. Castaño et al. demonstrated that scaffolds loaded with microRNA mimics and inhibitors augment bone growth and vascularization [222]. Many bone defects occur in the settings of infection, such as osteomyelitis, or contamination, such as trauma. Although antimicrobial scaffolds remain theoretical in clinical practice, they would likely decrease the rate of infections in implanted scaffolds used to repair bone defects. Polymers, peptides, carbon materials, metals, ceramics, and glass with inherent antimicrobial properties can be combined with antibiotics. Specifically, chitosan, cellulose, chitin, ponericin, SF, graphene oxide, Ag, gold (Au), Zn, Sr, and Mg, among many others, are materials that have been shown to have antimicrobial effects. Furthermore, scaffolds can be engineered to release antibiotics in a controlled fashion. Minocycline, chlorhexidine, berberine, doxycycline, and rifampicin can be directly loaded onto scaffolds. These antibiotics help to inhibit biofilm formation by bacteria. The main concerns for the translation of antimicrobial scaffolds to clinical practice are toxicity and antibiotic resistance [223]. As mentioned previously, scaffolds embedded with growth factors also often have immunomodulatory effects through the M2 polarization of macrophages. IL-4, IL-10, and IL-13 cytokines induce the M2 phenotype of macrophages, which is involved in wound healing and tissue repair. CaP nanoparticles, HAp nanoparticles, and graphene oxide nanoparticles all mediate an inflammatory or activated immunomodulatory microenvironment that recruits cytokines and macrophages to bone defects [224,225,226].

**Figure 6:**
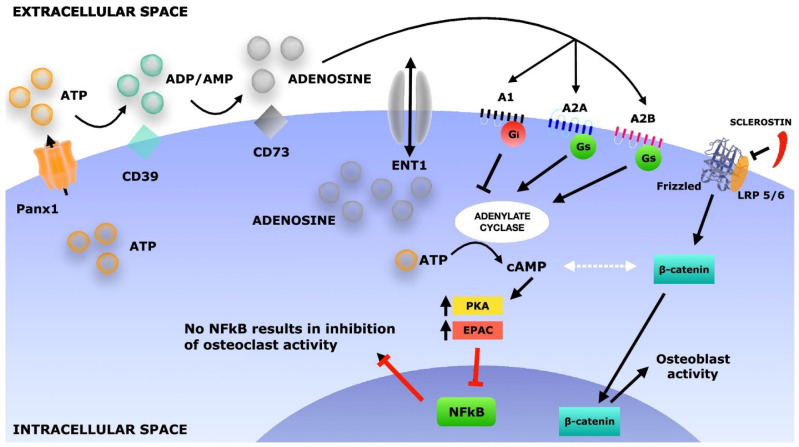
Schematic depicting adenosine receptor activation. Reprinted with permission from ref. [241]. Copyright 2019 Elsevier Ltd.

As previously mentioned, an established and widely used bioactive molecule is recombinant human bone morphogenetic protein 2 (rhBMP-2), which is involved in committing multipotent stromal cells toward an osteogenic lineage that in turn promotes the formation of new bone [227,228,229]. rhBMP-2 is approved for use in treatment of long bone fractures; however, clinical studies have demonstrated critical side effects pertaining to its use. These include vertebral osteolysis, ectopic bone formation, radiculitis, and the stimulation of cancer growth, to name a few [230,231,232]. As such, researchers are actively investigating alternative bioactive molecules for bone regeneration, including but not limited to adenosine receptor agonists (Figure 6).

Adenosine is an extracellular purine generated by all cells from the hydrolysis of adenine nucleotides. It has been recognized for its physiological functions through the activation of cell-surface receptors for over a century. Investigators are now beginning to understand its effects on bone tissue formation, which has emerged as a key metabolic pathway that can contribute to various phases of bone hemostasis and regeneration. Dipyridamole (DIPY) is one such biological compound that serves as an indirect adenosine agonist. Historically, used as a vasodilatory and antiplatelet agent, it acts to increase extracellular adenosine by inhibiting the equilibrative nucleotide transporter-1 (ENT-1) and prevents the degradation of adenosine [233]. As such, DIPY administration has been shown to induce the agonism of the A_2A_ receptor, which then stimulates ECM formation [234,235]. There were previous concerns that adenosine signaling using DIPY could produce a pro-inflammatory response, leading to fibrosis and malformation. Conversely, however, A_2A_ receptor stimulation has been shown to produce anti-inflammatory responses and recent studies utilizing DIPY have not reported any ectopic bone formation [236,237,238]. In fact, extensive preclinical models have been published in recent years, demonstrating the efficacy of DIPY in serving as an alternative to other more commonly used growth factors like rhBMP-2 [238,239,240,241,242,243].

### 2.5. Incorporation of Stem Cell into Scaffolds

MSCs can differentiate into various cell lineages and are also able to self-renew [244,245,246]. This differentiation potential can be controlled by regulatory genes capable of inducing progenitor cells to differentiate into a specific cell lineage [244,245]. This may encompass mesodermal, ectodermal, or endodermal lineages, such as osteogenic, chondrogenic, neuronal, muscular, or even hepatic cells, under specific in vitro conditions [244,247,248]. In addition to transcription factors being capable of inducing the differentiation of this cell type [244], it is also important to highlight the significant role of growth factors and induction chemicals in signaling, as well as the existence of a microenvironment built with biomaterial scaffolds capable of providing suitable conditions for proliferation and differentiation [244,249,250].

MSCs can be obtained from various tissues, such as the umbilical cord, endometrial polyps, menstrual blood, bone marrow, and adipose tissue, among others [244,251,252]. The utilization of this cell type has shown promising results in the repair and regeneration of a variety of tissues. Such elevated therapeutic potential can be attained due to several distinct attributes, including their ease of isolation and expansion in cell cultures, paracrine effects, immunomodulatory potential, migratory behavior, and multipotency [250]. They are an excellent therapeutic option for tissue regeneration, given the ease and reproducibility of their isolation, their high potential for expansion, and their capacity for molecular adaptations through biomedical engineering [250].

**Figure 7:**
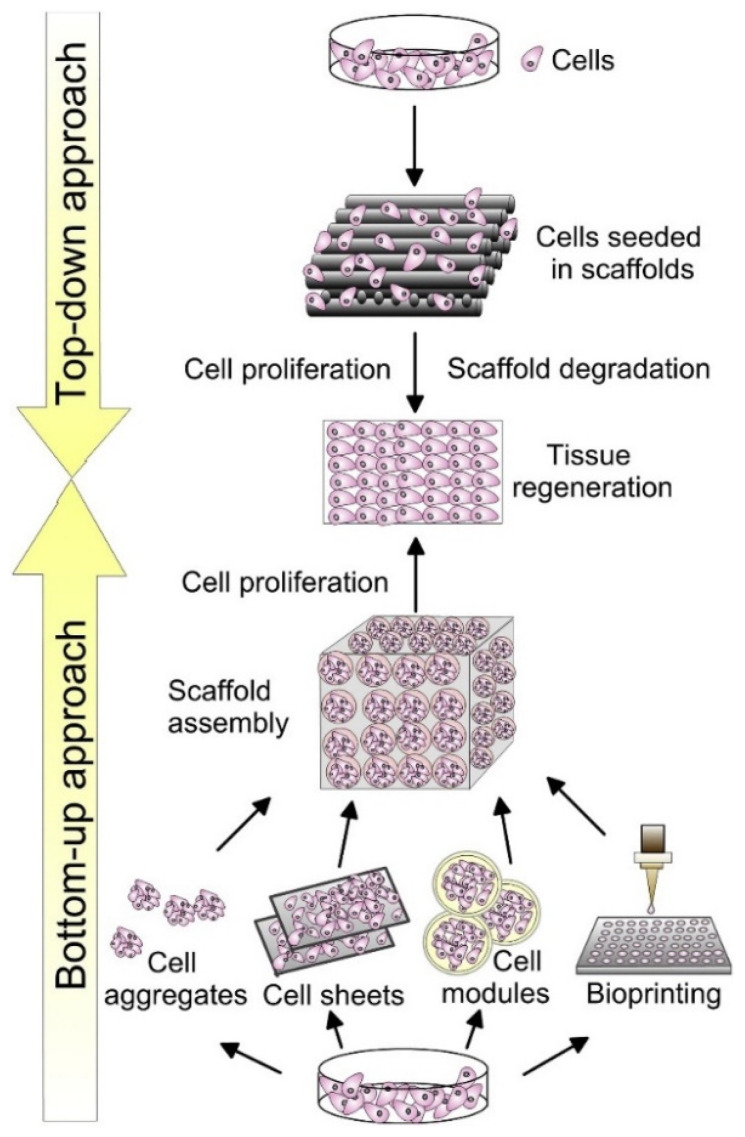
Representative schematic showing bottom-up and top-down strategies for tissue regeneration by using cell-laden scaffolds. Reprinted from ref. [262].

The incorporation of stem cells into scaffolds has emerged as a transformative approach in tissue engineering. Stem cells possess the unique ability to self-renew and differentiate into various other types facilitating the repair of defect sites. When combined with scaffolds, stem cells can enhance the biological activity and functionality of the construct, particularly beneficial in bone and joint surgery. The effective seeding of stem cells onto scaffolds is critical for ensuring optimal cell distribution, survival, and integration [253]. Several techniques have been developed to enhance the efficiency and uniformity of cell seeding, each with its own advantages. However, cell seeding can be broadly classified into two types, namely the bottom-up and the top-down approaches (Figure 7).

In the top-down approach, static seeding is one of the simplest approaches, where a cell suspension is pipetted directly onto the scaffold and allowed to attach through gravity and surface adhesion. While conveying ease of use, this method often results in nonuniform cell distribution, with most cells confined to the scaffold surface [254]. Dynamic seeding techniques aim to improve cell distribution throughout the scaffold. One common dynamic method is spinner flask seeding, where the scaffold is suspended in a flask containing a cell suspension that is continuously stirred. This creates a fluid flow that helps cells penetrate deeper into the scaffold structure. However, this can still lead to higher cell densities on the scaffold periphery [255,256]. Vacuum-assisted seeding uses negative pressure to draw the cell suspension through the scaffold pores. This technique has shown promise for achieving more uniform cell distribution, especially in hydrophobic scaffolds like a hyaluronan-based biodegradable polymer. Studies have reported over 90% cell retention after 24 h using this method [256]. Perfusion seeding involves continuously flowing a cell suspension through the scaffold using a bioreactor system. This provides better nutrient transport and can result in improved cell viability and distribution compared to static methods [253]. Lastly, centrifugal seeding utilizes centrifugal force to drive cells into the scaffold pores. This technique has demonstrated potential for enhancing cell penetration and distribution in porous scaffolds [255].

In the bottom-up approach, cell encapsulation, similar to biomolecule encapsulation, involves entrapping stem cells within a protective matrix or carrier system, which can then be incorporated into or onto a scaffold. One of the most common approaches is hydrogel encapsulation, where stem cells are suspended in a hydrogel precursor solution that is subsequently cross-linked to form a three-dimensional network around the cell [257]. Common hydrogels used in this method include alginate, COL, fibrin, and synthetic polymers like polyethylene glycol (PEG) [258]. This method allows for uniform distribution of cells throughout the scaffold, in addition to providing a biomimetic microenvironment for encapsulated cells. The hydrogel matrix protects the cells from mechanical stress and can be designed to mimic the ECM of the target tissue, potentially enhancing cell survival [258,259]. Specifically for bone and joint surgery applications, encapsulated MSCs have shown promise in forming new bone and cartilage [257]. Furthermore, studies have demonstrated that encapsulated MSCs can enhance bone formation when the capsules are loaded with BMP [257]. One of the key benefits is the sustained release of cells over time, leading to better tissue regeneration outcomes relative to traditional cell seeding methods [258]. However, it is important to note that the success of this method is dependent on various factors, including encapsulation material, capsule size, cell density, and the specific requirements of the target tissue [259]. The optimization of these parameters is crucial for ensuring cell viability, proper differentiation, and effective tissue regeneration in bone and joint surgical applications.

When we associate MSCs with bone tissue, it is known that this cell type can directly influence bone homeostasis [250]. The key factor in bone homeostasis is the balance between bone-forming cells (osteoblasts) and bone-resorbing cells (osteoclasts), and MSCs are widely recognized as precursors of osteoblasts [250]. Their action ranges from the frequency of generating new osteoblasts to their ability to produce factors affecting the osteoblast/osteocyte balance, such as receptor activators of nuclear factor B ligand (RANKL) and osteoprotegerin [250]. When it comes to cartilaginous tissue, it is known that the use of MSCs in repairing damage from cartilage defects and osteoarthritis (OA) is limited, given that many of these joint issues are age-related [250]. In most cases of joint damage, replacement with a prosthetic joint is necessary [250]. The initial literature studies indicate that the localized injection of autologous, culture-expanded chondrocytes seems to offer benefits for cartilage repair [250,260]. In contrast, tissue culture-expanded autologous MSCs in COL gel were injected into the knee joints of 24 patients with osteoarthritis, and no statistically significant benefit was observed, necessitating future studies to investigate the underlying factors [250,261].

### 2.6. Biomimetic Decellularized Tissue Scaffolds

Biomimetic decellularized tissue scaffolds are multidimensional structures that contain ECM proteins and growth factors and are designed to provide a framework for native tissue regeneration [263]. Decellularized biomimetic tissue scaffolds have the potential for regeneration of biologic tissues that are difficult to reconstruct or mimic. For example, Liu et al. showed that decellularized annulus fibrosus matrix combined with chitosan hydrogel had promising results in the regeneration of annulus fibrosus defects in rats [264]. In addition to annulus fibrosus tissue, tendons are an example of another tissue that has proven difficult to regenerate with synthetic scaffolds. As an example, Zhao et al. functionalized a 3D poly(l-lactide) porous scaffold with porcine-derived decellularized tendon matrix [265]. In another study, He et al. functionalized a PCL–laponite nanofiber membrane with decellularized ECM with excellent bone regeneration results in vivo [266]. The study showed that the nanofiber membranes functionalized with decellularized ECM improved mechanical properties, cytocompatibility, and osteoblast proliferation and differentiation [266]. In summary, scaffolds can be functionalized with a decellularized tissue matrix to elicit more favorable in vivo outcomes.

## 3. Advanced Applications

### 3.1. Multilayered Scaffolds

Cartilage damage is a prevalent medical condition for which various treatments have been developed. The goal of restorative procedures is to overcome the limitations of traditional approaches and restore the joint surface with hyaline tissues [267,268]. The main challenge in osteochondral repairs is the formation of the interface between cartilage and bone and integration with the original tissue. Usually, a multistage graft that mimics the structure and composition of osteochondral tissue is used to repair cartilaginous defects. Nie et al. manufactured a multistep graft using cartilage hydrogel and sintered PLGA microsphere structure bonded to ECM of endogenous fibrotic cartilage [269]. The graft showed gradient migration and integration from the cartilage layer to the subchondral bone layer, where the tissue quality is better, with efficient repair in a rabbit knee defect model [269]. In another study, Wang et al. developed a two-layered acellular osteochondral matrix (AOM) with a natural osteochondral–biomimetic microenvironment and an interface that enabled tissue-specific healing of osteochondral tissue [67]. The AOM scaffold was made with an ultraviolent laser and a decellularization technique, which facilitated adequate room for cell loading with favorable pore size [67]. A gelatin–methacryloyl (GelMA) hydrogel was used as a cell carrier to improve the efficiency and homogeneity of cell loading. In vitro results showed the AOM scaffold could effectively regulate BMSC differentiation by activating chondrogenic/osteogenic pathways [67]. An in vivo study revealed that combining AOM with a BMSC-loaded GelMA hydrogel successfully repaired osteochondral defects in the rabbit knee joint model, showing potential for clinical translation [67]. Shang et al. fabricated a nanotextured SF-CS/HAp nanowire, a strong bilayer structure, for osteochondral repair [76]. The bilayer was constructed using alcohol-induced β-sheet formation as a physical cross-linker. The osteochondral healing ability was evaluated by cultivating bone marrow MSCs in vitro and constructing a rat osteochondral defect model in vivo [76]. The in vivo results showed that the biomimetic bilayered construct significantly promoted new cartilage formation and subchondral bone remodeling in osteochondral defect models after implantation [76].

Advances in scaffold production and stem cell engineering have led to advances in the creation of composite tissues such as osteochondral tissue, but new approaches are needed to improve these results. Kang et al. manufactured a monolithic three-layered scaffold with different depths of pore architecture and a mineral medium to construct osteochondral tissues in vivo [270]. The three-layer scaffold contained a biomineralized base layer that mimics the microenvironment of bone containing CaP, a cryogel middle layer with anisotropic pore architecture, and a hydrogel top layer [270]. The bottom layer was kept cell-free, while the top two layers were loaded with cells before implantation [270]. When implanted in vivo, the three-layered scaffolds formed osteochondral tissue with a lubricin-rich cartilage surface [270]. In addition, Gegg et al. focused on the regeneration of zonal cartilage using spatially structured microribbon (μRB) hydrogels that mimicked the natural zonal organization of cartilage [271]. Three-layer hydrogel production involved sequentially adding hydrogel precursor solutions to a cylindrical mold and exposing them to UV light for polymerization [271]. To form μRB, Teflon sheets with concentric holes were filled layer-by-layer with μRB compounds and compressed before UV irradiation [271]. Overall, the study showed that the rapid development of cartilage mimics a compression modulus within 21 days, demonstrating a significant advance in cartilage regeneration techniques [271]. Another research study by Qiao et al. designed a tri-layered scaffold to replicate the spatial variations in osteochondral tissue’s ECM and COL fiber architecture [69]. The design combined a polymer of PCL and PEG with a gel-based hydrogel, incorporating MSCs and growth factors for layer-specific induction [69]. The scaffold’s mechanical strength was enhanced by the inclusion of poly(ε-caprolactone) and poly(ethylene glycol) (PCEC) fibers [69]. The experimental results showed the differentiation of MSCs into chondrogenic and osteogenic lineages, highlighting its potential for osteochondral regeneration [69].

Hybrid 3D scaffold printing has the potential to improve on the disadvantages posed by any single 3D scaffold printing method by combining the advantages of both synthetic polymers and natural polymers. Hung et al. described a hybrid 3D scaffold incorporating β-TCP and pluronic F-127, which demonstrated porous microstructural features with bone tissue ingrowth and mechanical stability [272]. Hydrogel and 3D-printed hybrid scaffolds have also been used in preclinical models of AC regeneration [273]. In vivo bone functions include, but are not limited to, bone formation, bone resorption, mechanical support and movement, calcium and phosphate cycling and homeostasis, and bone marrow storage [274]. Thereby, when a native bone is lost, its effective replacement inherently demands not only the biological and mechanical functions but also the immunomodulatory, vascular, and homeostatic functions of bones and joints. Advanced scaffold function in therapeutic modalities will be essential in treating bone infections, degenerative diseases, and cancer. Although these advanced technologies (summarized in Table 1) have not yet been fully clinically implemented, they are exciting avenues for expanding the effectiveness of bone scaffolds.

### 3.2. 4D Printing

**Figure 8:**
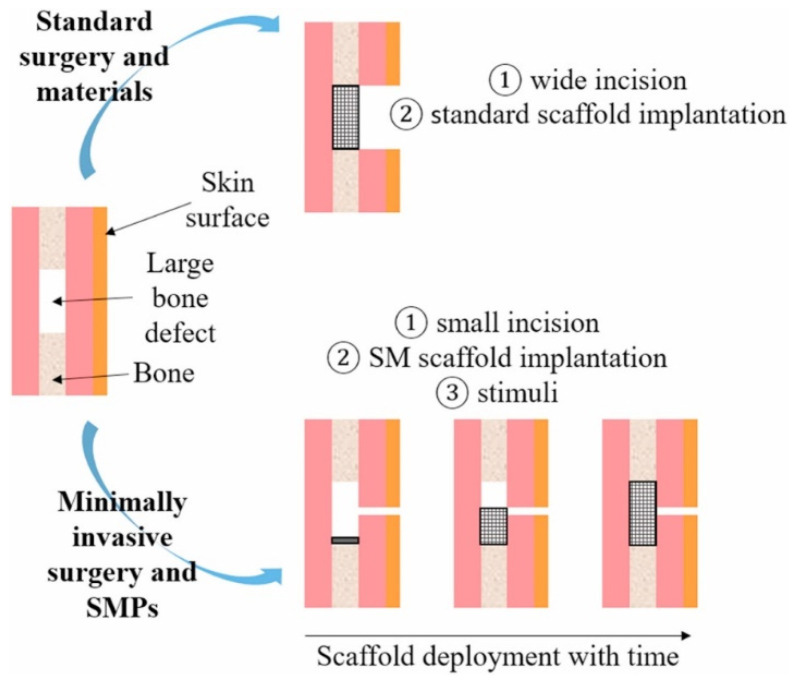
Example of the interest of using shape memory scaffolds for minimally invasive implantation into large bone defects. Reprinted with permission from ref. [192]. Copyright 2021 Elsevier Ltd.

The introduction of four-dimensional (4D) printing has significantly enhanced the capabilities of additive manufacturing. This technology enables programmable morphological transformation of 3D-printed structures in response to external stimuli [277,278,279]. In the domain of 4D printing, polymers are most commonly utilized owing to their low cost, variety, ease of availability, and more importantly their ability to be synthesized into dynamic and responsive structures that can adapt to their environment [280,281,282,283]. The 4D printing of polymers is particularly relevant in bone tissue engineering enabling the production of scaffolds that provide enhanced fit and improved functionality relative to traditional one-size-fits-all solutions. Taken one step further, the compact and deployable design of 4D-printed scaffolds has also been shown to be a viable option in minimally invasive surgical procedures [284,285]. For instance, a 4D-printed scaffold can be inserted through a small incision and subsequently expanded or changed in shape in situ, reducing the need for large, open surgeries, thereby expediting patient recovery (Figure 8) and reducing healthcare costs (associated with longer recovery and healing time) and improved patient outcomes [278,286].

Various external factors such as humidity, temperature, light, electricity, magnetism, and pH have been reported to facilitate shape memory behavior [287]. Among 4D-printed biomaterials that respond to changes in humidity, two previously studied moisture-responsive materials include cellulose tissue scaffolds and alginate/hyaluronic acid hydrogels. In response to water, tube-shaped scaffolds have been shown to swell and fold in on themselves, creating a change in shape and size [288,289,290]. Thermo-responsive materials change physical properties or function in response to changes in temperature. For example, poly(N-isopropylacrylamide) is rendered hydrophobic with an increase in temperature, specifically over a critical temperature of 36 °C. Conversely, temperature transitions below the critical temperature cause the polymer to become hydrophilic [287].

Near-infrared light and UV radiation can also induce structural changes in light-responsive shape memory polymers. However, UV radiation has been shown to induce DNA damage. Photo- and/or thermo-sensitive materials include thermoplastics like PLA, and other conjugated polymers. In a recent study, Choudhury et al. developed an extrusion based 4D-printed polylactide-co-trimethylene carbonate scaffold nanoengineered with polydopamine nanoparticles that expanded under near-infrared radiation to fit critical size cranial bone defects in rabbits, with promising results [275]. Of note, Wang et al. utilized photothermally responsive scaffolds with near-infrared radiation to fill irregular bone defects [276]. Some polymers like Hydroxylbutyl methacrylated chitosan are both thermo- and photo-responsive, undergoing thermal and photo cross-linking with changes in temperature and light [291].

Electrically reactive biomaterials include polythiophene, polyaniline, and polypyrrole, among others [292]. Chen et al. demonstrated that 4D-printed piezoelectric scaffolds can provide time-dependent electrical stimulation, essentially simulating the ideal bone tissue electrical microenvironment and promoting bone regeneration [293]. Similar to thermo-responsive materials, electroactive and magnetic hydrogels also function through expelling and attracting water through hydrophobic and hydrophilic interactions [291,294]. The binding of metal ions or changes in pH can stimulate changes in chemically reactive materials. Lastly, the binding of antibodies and enzymes in the microenvironment can also stimulate the transformation of a structure printed with a biologically reactive material [291].

### 3.3. Clinical Trials

Most scaffolds currently being used clinically in orthopedics are ECM and COL scaffolds. In 2016, Murray et al. described the Bridge-Enhanced Anterior Cruciate Ligament Repair (BEAR) procedure in humans, where a bioactive ECM scaffold was used to augment anterior cruciate ligament repair with low rates of adverse events but a small sample size [295]. At the 2-year follow-up, the bioactive scaffold demonstrated noninferiority compared to autologous anterior cruciate ligament reconstruction [296]. Few clinical trials have utilized 3D-printed synthetic and bioceramic scaffolds in orthopedics. Of note, a Phase IIa study of autologous bone marrow-seeded β-TCP scaffold for proximal humerus fractures showed no adverse effects but failed to show significant improvement in fracture healing [297]. A case series of a synthetic nanofiber resorbable scaffold composed of PCL and poly-L-lactic-co-ε-caprolactone (PLCL) for rotator cuff repairs demonstrated a 91% healing rate with no adverse effects [298,299]. However, the majority of synthetic or bioceramic scaffolds being used in clinical trials are used for periodontal and craniofacial defects rather than long bones and joints.

Critical-size bone defects are generally at least 1 cm bone defects that are not expected to heal without surgical intervention. However, there is no universally accepted definition. Current clinical treatment options for critical-size bone defects include cancellous grafts, reconstruction with a cement spacer, vascularized bone grafts, and bone transport, all with or without antibiotics [300]. Tissue-engineered bone and joint scaffolds are potential solutions to the reconstruction of critical-size bone defects when conventional methods fail. A Phase IIa clinical trial of a PCL-TCP printed scaffold along with cortiocoperiosteal tissue during the reconstruction of lower limb bone defects is currently ongoing [301]. A small clinical trial of a Trumatch PCL and HAp graft for segmental long bone defects is also currently in its early stages (ClinicalTrials.gov Identifier: NCT05668182). Few, if any, previous clinical trials of 3D-printed scaffolds to treat critical-size bone deficits have been performed. As Laubach et al. emphasized, there is a significant gap between the extensive preclinical studies on the treatment of bone defects and clinical application in surgery [302]. A summary of clinical trials using 3D-printed synthetic and bioceramic scaffolds for bone and joint regeneration is provided in Table 2.

### 3.4. Critical Summary of Materials and Production Methods for Bench-to-Bedside Translation

There is a significant discrepancy between the advancements in biomaterials for bone regeneration and the production methods specifically in light of guidelines set forth by regulatory authorities. Nonetheless, based on the considerable potential of advanced biomaterials, it is expected that a growing number of researchers/companies will seek regulatory approval for their novel materials to treat bony defects. While the results of preclinical experiments have shown promising outcomes using advanced materials and production methods, addressing potential clinical issues pertaining to such combinatorial strategies for the treatment of bone defects remains a concern. To elaborate, while conventional regulatory routes are primarily intended for mass-produced remedies rather than patient-specific solutions, the inclusion of living cells, and the variety of material syntheses and manufacturing process techniques add to the level of complexity. Among all the manufacturing methods investigated, 3D printing has shown considerable promise for the synthesis of such next-generation, customizable, high-fidelity tissue engineering devices. However, as previously stated, each modality of 3D printing has its own advantages/limitations and presents potential for improvement. The future of 3D printing for bone regeneration could not only potentially entail exploring novel materials/bioinks that incorporate sophisticated biomaterials and growth factors to augment cell growth and expedite vascularization, but also examining hybrid 3D printing systems to surpass the constraints of any single 3D printing technique and introducing 4D printing as a novel concept to address the current obstacles faced by 3D printing products for clinical applications.

A fundamental limitation of medical 3D printing modalities is the lack of defined standards and quality control protocols to assess the quality and uniformity of the build and its biological effects. Notwithstanding, the initiation of the first clinical trial for 3D-printed devices in recent years has heightened the need for revised regulations on the implementation of this technology in clinical settings. Many international regulatory agencies, such as the Food and Drug Administration in the USA, the European Medicines Agency in Europe, and the Therapeutic Goods Administration in Australia, are investigating potential modifications to their regulations for medical 3D printing. While these organizations impose rigorous classification and standardization criteria, the classification of 3D-printed devices is further complicated due to their characteristics to serve as medical devices and/or biologics, which are each governed by distinct regulatory procedures. Moreover, bioprinting of tissue engineering devices could potentially be compromised through variations in material composition and printer calibration, to name a few. In addition, the inclusion of living cells gives rise to notable safety issues such as the possibility of immunogenic responses, and risk of disease transmission. As such, continued surveillance of adverse events and long-term results are essential to ascertain the effectiveness and safety of 3D-printed devices.

## 4. Conclusions

In summary, bone and joint tissue engineering, specifically functional scaffolds, has advanced significantly in recent years to attempt to fill the need for a reliable, widely available, and cost-effective substitute for bone and cartilage. However, a significant gap remains between in vivo and in vitro studies of bone and joint scaffolds and their implementation in clinical practice for the treatment of long bone and joint defects. The main challenges that exist for the 3D printing of implantable bone and joint scaffolds include a lack of standards for implantable biomaterials, a lack of translational research implementing 3D-printed materials in the clinical setting, and a lack of adequate replication of porous microstructures for mechanical, immunological, and biochemical properties [306]. Most of these factors are inter-related and in part prohibitive of the others. The timed release of bioactive materials from scaffolds, stem cells seeded onto scaffolds, tri-layered scaffolds, and hydrogels are only a few of these advances, which can potentially, prospectively bridge the gap between preclinical and clinical research. Materials, printing technologies, and additive components are becoming more effective at modeling 3D scaffolds into functional—in terms of mechanics, immune function, osteogenesis, and homeostatic properties—printed grafts of bone and joints. However, despite the aforementioned clinical advancements, the search for the optimal biomaterial or scaffold continues and warrants further investigation.

## Figures and Tables

**Table 1:** Overview of advanced applications of scaffolds.

Scaffold Type	Tissue	Printing Method	Materials	Results	Source
Multiphasic graft	Osteochondral defect	SLS, bonding of cartilage hydrogel, and decellularization technique	Cartilage hydrogel and sintered PLGA microsphere structure bonded to ECM of endogenous fibrotic cartilage	Gradient migration and integration from the cartilage layer to the subchondral bone layer, with efficient repair in a rabbit knee defect model. Following decellularization, the tissue repair efficacy of the graft decreased.	[269]
Double-layered graft	Osteochondral defect	UV laser and decellularization technique	Acellular osteochondral matrix (AOM) with a natural osteochondral–biomimetic and gelatin–methacryloyl (GelMA) hydrogel	In vitro results showed the AOM scaffold could effectively regulate BMSC differentiation by activating chondrogenic/osteogenic pathways. In vivo results revealed that combining AOM with BMSC-loaded GelMA hydrogel successfully repaired osteochondral defects in the rabbit knee joint model.	[67]
Bilayer membrane	Osteochondral defect	Alcohol-induced β-sheet formation	Nanotextured SF-CS/HAp nanowire	In vivo, the biomimetic bilayered construct significantly promoted new cartilage formation and subchondral bone remodeling in osteochondral defect models after implantation.	[76]
Monolithic three-layered scaffold	Osteochondral defect	Stepwise polymerization in poly(ethylene glycol)-diacrylate, N-acryloyl 6-aminocaproic acid, and modified simulated body fluid solutions	Biomineralized cell-free base layer that mimics the microenvironment of bone containing CaP, a cryogel middle layer with anisotropic pore architecture, and a hydrogel top layer	When implanted in vivo, the three-layered scaffolds formed osteochondral tissue with a lubricin-rich cartilage surface.	[270]
Tri-layered microribbon (μRB) scaffold	Osteochondral defect	Hydrogel precursor solutions sequentially added to a cylindrical mold and exposed to UV light for polymerization	Teflon sheets with concentric holes are filled layer by layer with μRB compounds and compressed before UV irradiation. Aligned μRB can also be added as a fourth layer before UV irradiation.	Overall, the study showed that the rapid development of cartilage mimics the compression modulus within 21 days, demonstrating a significant advance in cartilage regeneration techniques.	[271]
Tri-layered stratified scaffold	Osteochondral defect	Melt electrowriting	MSC-laden gelatin methacrylamide (GelMA) hydrogel with zone-specific growth factor delivery combined with melt electrowritten triblock polymer of poly(ε-caprolactone) and poly(ethylene glycol) (PCEC) networks	The experimental results showed the differentiation of MSCs into chondrogenic and osteogenic lineages, highlighting their potential for osteochondral regeneration.	[69]
Hybrid 3D scaffold	Bone defects	Extrusion-based bioprinting	β-TCP and thermoreversible pluronic F-127	Demonstrated porous microstructural features with bone tissue ingrowth and mechanical stability.	[272]
4D-printed photothermal responsive scaffold	Irregular bone defects	Extrusion-based bioprinting	Polylactide-co-trimethylene carbonate scaffold nanoengineered with polydopamine nanoparticles	Near-infrared light induced the expansion of the scaffold to fit critical size cranial bone defects in rabbits, with promising results.	[275]
4D-printed photothermal responsive scaffold	Irregular bone defects	Extrusion-based bioprinting	Black phosphorus nanosheets and osteogenic peptide incorporated into β-tricalcium phosphate/poly(lactic acid-co-trimethylene carbonate) (TCP/P(DLLA-TMC)) nanocomposite scaffolds	Near-infrared light induced expansion of the scaffold to fit critical size cranial bone defects in rats. Osteogenesis further improved through pulsed peptide release from the scaffold.	[276]

**Table 2:** Summary of clinical trials using 3D-printed synthetic and bioceramic scaffolds for bone and joint regeneration from 2014 to 2024.

Cells	Scaffolds	Condition	Patients	Control	Outcome	Year	Source
Plasmid DNA Encoding Vascular Endothelial Growth Factor (VEGF)	COL-HAp Composite	Maxillofacial Bone Defects	12	_	Consolidation of previous distal nonunion, but unsuccessful proximal nonunion	2014–2017	NCT02293031 [303]
Bone Marrow Aspirate Concentrate (BMAC)	HA	AC Defect	200	Microfracture	No preliminary results	2016–2026	NCT02659215 [304]
Autologous Bone Marrow Mononuclear Cells	β-TCP	Proximal Humerus Fracture	56	β-TCP only	Radiographic bone healing and DASH score did not differ between two groups	2016–2019	NCT02803177 [297]
Autogenous Bone	HAp, PCL	Periodontal Defect	20	Autogenous Bone + COL Membrane	Unknown Status	2017–2019	NCT03232788
MSCs	PLGA	Aneurysmal Bone Cysts	4	_	Unknown Status	2018–2020	NCT03066245
Plasmid DNA Encoding VEGF, Autogenous Bone	Octacalcium Phosphate (OCP)	Nonunion of Long Bones	20	Bone reconstruction with Autogenous Bone	Unknown Status	2020–2022	NCT04705857
Bone marrow aspirate concentrate (BMAC)	PCL	Alveolar Bone Defect	7	_	Unknown Status	2022–2022	NCT05241548
Corticoperiosteal Flap	PCL-TCP	Lower Limb Critical Size Defects	10	_	No Preliminary Results	2023–2028	ACTRN12620001007921 [301]
No Cells	Bilaminar Chitosan	Sellar Floor Repair	1	_	Normal vision, no evidence of lesion, no complications	2017–2019	NCT03280849 [305]
No Cells	TiO2	Alveolar Bone Defect	10	_	Unknown Status	2021–2023	NCT06269497
No Cells	PCL, HAp	Long Bone Defect	5	_	No Preliminary Results	2021–2026	NCT05668182
No Cells	HAp, β-TCP Polydioxanone	Alveolar Bone Defect	60	Extraction Only or Extraction + Laser	Unknown Status	2022–2023	NCT06150456
No Cells	Mg Calcium Silicate	Alveolar Bone Defect	5	_	No Preliminary Results	2022–now	NCT05743452
No Cells	HAp, β-TCP Polydioxanone	Alveolar Bone Defect	60	Extraction Only or Extraction + Laser	No Preliminary Results	2023–2024	NCT06164626

## Data Availability

No new data were created or analyzed in this study. Data sharing is not applicable to this article.

## Abbreviations

AC	Articular cartilage
Ag	Silver
AM	Additive manufacturing
AOM	Acellular osteochondral matrix
BMSC	Bone marrow stromal stem cell
CaP	Calcium phosphate
CNF	Carbon nanofiber
COL	Collagen
CPC	Calcium phosphate cements
CS	Chondroitin sulfate
Cu	Copper
DCH	Dodecahedron
DG	Dense granules
DICOM	Digital imaging and communications in medicine
ECM	Extracellular matrix
F	Fluoride
FDsI	Full dense surface layer
Fe	Iron
G	Grid
GAG	Glycosaminoglycan
GelMA	Gelatin–methacryloyl
HA	Hyaluronic acid
HAp	Hydroxyapatite
HCG	Honeycomb granules
La	Lanthanum
LENS	Laser-engineered net shaping
MBG	Mesoporous bioactive glass
Mg	Magnesium
Mn	Manganese
MSC	Mesenchymal stem cell
MZS	Multizonal scaffold
NC	Network connectivity
OCT	Octet-truss
PA	Polyamide
PCEC	Poly(ε-caprolactone) and poly(ethylene glycol)
PCL	Polycaprolactone
PDLLA	Poly(D,L-lactide)
PDMS	Polydimethylsiloxane
PDsI	Partially dense surface layer
PEG	Polyethylene glycol
PET	Polyethylene terephthalate
PGA	Polyglycolic acid
PLA	Polylactic acid
PLGA	Poly-L-lactic-co-glycolic acid
PP	Polypropylene
PPF	Polypropylene fumarate
PS	Polystyrene
PU	Polyurethane
PVOH	Polyvinyl alcohol
SF	Silk fibroin
SLM	Selective laser melting
Ta	Tantalum
TCP	Tricalcium phosphate
Ti	Titanium
TPE	Thermoplastic elastomers
UV	Ultraviolet
VEGF	Vascular endothelial growth factor
Zn	Zinc
β-TCP	Beta-tricalcium phosphate
μRB	Microribbon
